# Moral dilemmas reflect professional core values of pharmacists in community pharmacy

**DOI:** 10.1111/ijpp.12490

**Published:** 2018-10-19

**Authors:** Martine Kruijtbosch, Wilma Göttgens‐Jansen, Annemieke Floor‐Schreudering, Evert van Leeuwen, Marcel L. Bouvy

**Affiliations:** ^1^ SIR Institute for Pharmacy Practice and Policy Leiden The Netherlands; ^2^ Division of Pharmacoepidemiology and Clinical Pharmacology Department of Pharmaceutical Sciences Utrecht University Utrecht The Netherlands; ^3^ Radboud University Medical Center Radboud Institute for Health Sciences (RIHS) Scientific Center for Quality of Healthcare (IQ Healthcare) Nijmegen The Netherlands

**Keywords:** community pharmacy, moral dilemmas, Netherlands, pharmacists, professional core values

## Abstract

**Objectives:**

The aim was to recognise the professional core values in the moral dilemmas of pharmacists in community pharmacy and to customise the descriptions of these values for community pharmacy practice.

**Methods:**

The narratives of 128 moral dilemmas, collected from Dutch PharmD students and early career pharmacists who experienced these dilemmas in practice, were qualitatively analysed. An expert panel deductively coded relevant portions of these narratives with the core values as formulated by the Royal Dutch Pharmacists Association. Other values that emerged were inductively coded and if possible used to further customise the respective core values.

**Key findings:**

The expert panel identified all four professional core values, that is, *commitment to the patient's well‐being* (117, 91.4%), *reliable and caring* (116, 90.6%), *pharmaceutical expertise* (72, 56.2%) and *responsibility to society* (30, 23.4%) in the 128 moral dilemma narratives. Thirteen other values that emerged in the analysis could all be used for the customisation of the professional core values in descriptions that better reflect community pharmacy practice.

**Conclusions:**

Professional core values were identified in moral dilemma narratives of pharmacists in community pharmacy and customised for their practice. These customised core values can enable pharmacists to better recognise moral dilemmas in practice. This can add to the advancement of the profession as a pharmaceutical care practice.

## Introduction

Historically, accuracy and safety in the compounding and dispensing of medicines have been the most important values for pharmacists.^1^, ^2^ Because pharmacists’ focus shifted from product to patient,^3^, ^4^, ^5^ the professional values of the profession need reformulation.^3^, ^6^, ^7^, ^8^, ^9^ Professional values are the foundation of a professional practice and are thus specific to a practice.^10^ The practice of pharmaceutical care implies that pharmacists take responsibility for definite outcomes of drug therapy that improve patients’ quality of life.^6^ To realise this practice, pharmacists should develop professional behaviour and a common professional identity that goes beyond their personal values and self‐interests.^10^, ^11^, ^12^ Therefore, clearly described professional values are needed to support pharmacists in this development.^13^, ^14^, ^15^, ^16^, ^17^

However, since the introduction of the ‘pharmaceutical care practice’ in the 90s, research on professional values in pharmacy is scarce.^6^ The following values have been suggested as foundational to pharmacy practice as well as any other healthcare practice: self‐determination, compassion, justice, respect for persons, commitment to integrity and ethical practice and commitment to excellence.^14^, ^16^ Qualitative studies on the understanding of ‘patient‐centred professionalism’ also emphasise that professional values are important to advance healthcare practices and to achieve definite positive health outcomes in patients.^15^, ^17^ In medicine and nursing, experts have raised awareness that professional values should be taught to starting professionals in order to create a shared culture of practice and stimulate a commitment to the profession's values.^12^, ^18^ Such a commitment may improve patients’ health outcomes, as was found in a recent study among nurses.^19^

Despite limited research, professional values of pharmacy practice appear, often implicitly, in pledges of professionalism. For example, in many countries, pharmacists take public oaths, mostly at the graduation ceremonies of pharmacy schools.^3^, ^20^, ^21^ These pledges are often inspired by oaths such as the Hippocratic Oath.^3^ The International Pharmaceutical Federation (FIP) recently reached consensus on a new oath based on existing documents^22^ to emphasise the professional commitment among pharmacists worldwide. In 2012 the Royal Dutch Pharmacists Association (KNMP) adopted a charter defining the professional core values through consensus with pharmacists who work in all different practices (e.g. community and hospital pharmacy, industry, research, development and government). These values included: (1) *commitment to the patient's well‐being*, (2) *reliability and care*, (3) *pharmaceutical expertise*, (4) *social responsibility* and (5) *professional autonomy*. These core values should guide every pharmacist, irrespective of the practice setting.^23^

Thus far, it has not been studied whether and how these professional core values of pharmacists play a role in their specific professional practices.^21^ Moreover, the professional values have not been defined for the individual practices of the pharmacy profession (e.g. community pharmacy, hospital pharmacy, industry and government).^14^ The awareness of professional values can help health professionals to make appropriate decisions and behave responsibly in their patients’ best interests.^24^ Therefore, the aim of this study was to recognise the professional core values in the moral dilemmas of pharmacists in community pharmacy and to customise the descriptions of these values for community pharmacy practice.

## Method

### Study design and setting

During classes on professionalism and pharmaceutical ethics in the pre‐ and postgraduate education for Dutch PharmD students and early career pharmacists, the students and pharmacists were trained to recognise moral dilemmas. The training included studying literature on pharmaceutical care practice, pharmacy ethics and pharmacists’ professional values, followed by presentations by the trainers to deepen understanding of these topics. Examples of moral dilemmas were discussed in small groups to learn how to recognise a core problem and the professional values involved therein. As an assignment, the students and pharmacists were asked to write a narrative of a moral dilemma they had experienced in community pharmacy practice. The narrative had to be written shortly after they had experienced the dilemma, and they had to describe their own preferred ethical stances and values explicit therein. On the basis of the various definitions in the literature,^25^, ^26^, ^27^, ^28^, ^29^ a moral dilemma was defined as *a situation in which there is a choice between at least two courses of actions, neither of which is obviously morally preferable*.

In our previous study, we took a stratified random sample of 128 narratives written in 2010–2012 and analysed the themes of the moral dilemmas described in these narratives.^30^ The same moral dilemma narratives were used for the present study.

### Identification and coding of values

The relevant portions of 128 written moral dilemma narratives that reflected the motives, arguments and considerations of the pharmacists were deductively coded with the core values of the Dutch Charter Professionalism of the Pharmacist; Foundation to act professionally and ethically.^23^ Although professional autonomy is included in this charter as a fifth core value, this value could not be analysed because the outcome of the moral dilemma was not always clearly described in all the narratives. See Table 1 (first column) for the four core values. Other values that emerged from the moral dilemmas, but were not covered by the charter's core values’ descriptions, were inductively coded (see Table 1).

**Table 1:** Professional core values customised for pharmacists in community pharmacy

**Dutch Charter professional core values**		**Identified OTHER values NOT covered by the charter description**		**Dutch Charter professional core values customised for pharmacists in community pharmacy**
**Commitment to the patient's well‐being**				**Commitment to the patient's well‐being**
Every pharmacist is directly or also indirectly involved in the patient's well‐being: as a direct care provider, as a compounder or developer of medicines or within the educational sector or regulations	**+**	Autonomy of the patient Self‐determination of patient Protect life	**→**	The pharmacist is **committed to the patient's well‐being**. This commitment includes **respecting the patient's preferences and values** and subsequently **facilitating shared decision‐making**. The pharmacist **respects the patient's right to self‐determination**
**Pharmaceutical expertise**				**Pharmaceutical expertise**
Like any other professional, the pharmacist also has specific expertise and competences that he can use to provide the best possible service to society. The expertise is related to the pharmacist's specific knowledge domains. It is systematically and frequently maintained				The pharmacist is a **competent expert who helps patients and doctors to optimise the effective and safe use of medicines**. The pharmacist's expertise emanates from specific knowledge of (patho)physiology, pharmacotherapy, pharmacokinetics, pharmacodynamics, pharmaceutics and health psychology
**Reliability and care**				**Reliable and caring**
Medicines in general are powerful substances. They can be highly effective, but at the same time unsafe. The quality assurance of the pharmacist's actions must therefore be beyond doubt	**+**	Professional collaboration with colleagues and other health professionals Privacy of the patient Being reliable within the pharmacist‐patient relationship Personal and professional integrity Adhering to rules and regulations Loyalty towards colleagues and other health professionals	**→**	Medicines can be highly effective, but at the same time carry risks of causing harm. **Quality assurance** by the pharmacist is therefore crucial. The pharmacist **acts meticulously and carefully** (e.g. compounding or dispensing medicines, counselling patients, monitoring medicine use and keeping patient records). The pharmacist **maintains a relationship of trust with the patient**. Moreover, the pharmacist **respects the patient's confidentiality**. The pharmacist **acts reliably within the collaboration with other health professionals**
**Social responsibility**				**Responsibility to society**
This core value emphasizes that the pharmacist's actions are efficient and transparent not only for the individual patient but also for society, and that the pharmacist feels a sense of responsibility for the social consequences of his actions	**+**	Sustainability of the pharmacy Trust in pharmacy practice Access to medicines Continuity of care	**→**	The pharmacist is **responsible for the societal consequences of his or her actions**. In order to **maintain patients’ and the public's trust** in the pharmacy practice and the healthcare system, the pharmacist **acts transparently and treats patients equally.** The pharmacist **guarantees access to pharmaceutical care and its continuity** by collaboration with other health professionals

An expert panel consisting of the first author (MK) and eleven senior practicing pharmacists performed the coding. The pharmacists of the expert panel were members of the ethics working group of the KNMP and were trained in a half‐day ethics course to identify (core) values. MK coded all narratives, and each other panellist coded twenty narratives. If consensus about coding was not reached, a third pharmacist from the research group (AF, MB or WG) was consulted. Coding and counting of values were facilitated by ATLAS.ti (version 7.5.17, GmbH, Berlin).

### Customisation of professional core values for community pharmacy

After coding, the research group independently analysed the ‘other values’ to either match them with the core values of the charter or categorise them separately. The matched ‘other values’ were used to revise the original description of each of the core values of the charter into descriptions more accordant with the practice of community pharmacy. This customisation expressed the considerations of the pharmacist when deliberating on how to proceed with the dilemma. Subsequently, the expert panel was consulted to reach consensus on these adapted core values descriptions.

### Ethics and confidentiality

The Medical Ethics Review Committee of the University Medical Centre Leiden concluded that the Dutch Medical Research Involving Human Subjects Act (WMO) was not applicable. All participants consented that their narratives could be used for the purpose of the study. Data that could give clues about the origin of dilemmas (e.g. names of patients, cities, pharmacies, pharmacists or physicians) were removed.

## Results

The 128 narratives were written by pregraduates (49%: 51% male #bib49% female) and postgraduates (51%: 39% male, 61% female).

In addition to the professional core values, thirteen ‘other values’ initially emerged. All these ‘other values’, however, could be matched with the core values as the research group and the expert panel viewed them as relevant additions (Table 1). This resulted in customised descriptions of the core values reflecting community pharmacy practice and in small adaptations in the names of two values (Table 1).

For example, the ‘other value’ *autonomy of the patient* was included in the core value commitment to the patient's well‐being. The ‘other value’ *protect life* was also seen as part of this core value. However, this ‘other value’ was not explicitly included in the customised description because a situation can exist in which a patient prefers care that aims to end his or her life (e.g. euthanasia) rather than to protect it.

The customised core values, in comparison to their original descriptions, were adapted to the greatest degree for *reliability and care* and *social responsibility*. The first value name was changed to *reliable and caring* in order to reflect the caring role of the pharmacist. This was motivated by the incorporation of identified other values listed under this core value (see Table 1), such as *being reliable within the pharmacist‐patient relationship* and *collaboration with other health professionals*. The term *responsibility to society was considered* more appropriate than *social responsibility* because it better reflects the responsibility that pharmacists expressed in the narratives to guarantee access to medicines and pharmaceutical care for all patients. Other identified values such as *trust in pharmacy practice, sustainability of the pharmacy* and *continuity of care* contributed to that consideration.

Although no ‘other values’ were incorporated into *pharmaceutical expertise* (see Table 1), the original description of this core value was adapted to the practice of community pharmacy based on the specific areas of knowledge that emerged from the narratives.

### Recognition of customised professional core values

**Figure 1:**
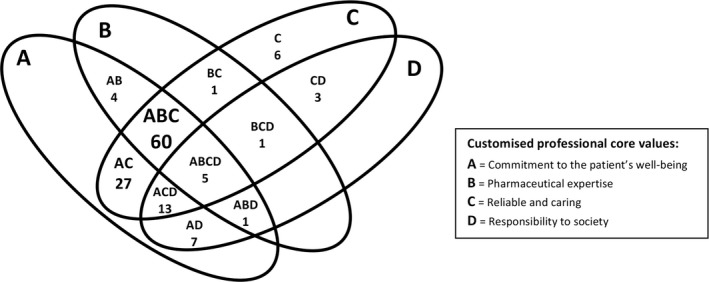
Recognition of (combinations of) customised professional core values in 128 moral dilemmas.

In the 128 moral dilemma narratives, the core values *commitment to the patient's well‐being* and *reliable and caring* emerged most prominently. These values were identified in 117 (91.4%) and 116 (90.6%) moral dilemma narratives, respectively. *Pharmaceutical expertise* (72, 56.2%) and *responsibility to society* (30, 23.4%) were less often identified. When the combinations of the identified core values were counted, the combination of *commitment to the patient's well‐being, reliable and caring* and *pharmaceutical expertise* (combination of A, B and C in Figure 1) appeared in the majority (60, 46.9%) of the moral dilemma narratives. Only in five (3.9%) moral dilemmas did all four core values (combination of A, B, C and D in Figure 1) emerge together. In six (4.7%) moral dilemma narratives, only one core value, *reliable and caring* (C in Figure 1)*,* was involved. In these dilemmas, different customised parts of that professional value played contextual roles.

Six examples of moral dilemmas (Tables 2, 3, 4) with coded quotes that represent the customised professional core values are available as online supplements.

**Table 2:** Moral dilemmas 1 and 2

**Moral dilemma 1 (PHARM‐1239)**	
A patient had been dispensed 10 tablets of ibuprofen 600 mg after a dental procedure the previous day. The patient returns for a refill and explains that she experienced only one hour's pain relief after one dose and had already finished her tablets. The pharmacist was uncertain on whether to dispense	
	**Customised professional core values:**
‘I wanted to help her get rid of her pain’	**Commitment to the patient's well‐being**
‘On the other hand, I suspected an infection as the effect of the ibuprofen should be longer than one hour. Hence, I referred her to the physician’	**Pharmaceutical expertise**
‘She didn't want this advice and angrily persisted in demanding ibuprofen’	**Reliable and caring** (*maintaining a relationship of trust with the patient*)

**Moral dilemma 2 (PHARM‐244)**	
A 50‐year‐old woman is overusing the laxative bisacodyl. She buys several boxes per week. The pharmacist had tried to discuss the overuse and the possible harm this could cause several times. The woman was never interested in discussing it. Hence, the pharmacist decided to no longer dispense the medicine and referred her to her physician to obtain a prescription. The next day she presented a prescription	
	**Customised professional core values:**
‘It is my task to inform the patient about the detrimental effects of overusing this drug’	**Commitment to the patient's well‐being** (*facilitating shared decision‐making*) **Pharmaceutical expertise**
‘Should I inform her physician about her overuse?’	**Reliable and caring** (*respecting patient's confidentiality*)

**Table 3:** Moral dilemmas 3 and 4

**Moral dilemma 3 (PHARM‐79)**	
Before closing time on Friday afternoon, a 12‐year‐old patient requested extra methylphenidate for the weekend. The prescriber could not be reached at that time. The boy needs the medication for chronic attention deficit hyperactivity disorder (ADHD). He revealed that his mother had used his medication. When the pharmacist contacted the mother, she confirmed that she was going through a rough time and had used her child's medication	
	**Customised professional core values:**
‘As there was no prescription and the boy's physician could not be reached, I had a dilemma: keeping with the law that forbids to dispense without a prescription…’	**Reliable and caring** (*acting meticulously and carefully*)
‘…my concerns about the possible harm to the mother using such a drug without having consulted a physician, and my responsibility for the child's health over the weekend’	**Commitment to the patient's well‐being**

**Moral dilemma 4 (PHARM‐263)**	
A patient had used paroxetine for five weeks. She asked the pharmacist for advice on how to discontinue the medicine as soon as possible because she suffered heavily from side effects	
	**Customised professional core values:**
‘She persisted in her request to discontinue “these chemicals”. She had tried to contact her physician, but he was on summer holidays and told me that she was not willing to consult another physician to discuss her problem’	**Commitment to the patient's well‐being** (*respecting the patient's preferences and values; respecting the patient's right to self‐determination*)
‘Motivating this patient to continue treatment seemed useless. However, making a scheme to discontinue the paroxetine without consulting her physician did not feel right either. We had previously agreed with the local physicians to closely monitor and counsel patients, starting with antidepressants to prevent them from stopping’	**Reliable and caring** (*acting reliably within the collaboration with other health professionals*) **Pharmaceutical expertise**
‘On the other hand, if I did not provide her with such a scheme, she might have stopped abruptly and experienced withdrawal symptoms’	**Commitment to the patient's well‐being** Reliable and caring (*acting meticulously and carefully*) **Pharmaceutical expertise**

**Table 4:** Moral dilemmas 5 and 6

**Moral dilemma 5 (PHARM‐1071)**	
The pharmacist noticed that a patient with diabetes II was using much more insulin, test materials and needles than prescribed. When the pharmacist asked the patient about it, he stated that this regimen was advised by the hospital physician. The pharmacist, however, had seen the letter from the hospital physician to the GP, which stated a much lower dose. The patient had family living abroad and regularly visited them. Both the pharmacist and the physician suspected that the patient probably provided relatives or friends there with insulin	
	**Customised professional core values:**
‘The patient used three times the dose requirements of insulin a person with this weight should use’	**Pharmaceutical expertise**
‘The earlier dispensed quantities had already cost Dutch society about 15.000 euros extra’	**Responsibility to society** (*responsible for the societal consequences of his or her actions*)
‘On the other hand, the patient actually needed medication. There might have been even other explanations for the overuse’	**Commitment to the patient's well‐being**

**Moral dilemma 6 (PHARM‐109)**	
A woman presents a prescription for the oncolytic melphalan for her father who lives in another country. The prescription is unclearly written and is in a foreign language. She explains that her father needs the drug but that it is not available in his country	
	**Customised professional core values:**
‘I doubted about dispensing because I had no clear information about the indication, nor the dose and quantity of the drug to dispense. The potential toxicity of the drug made me even more careful. Also I did not know this patient nor the prescriber, and therefore I did not have the relevant patient data in order to dispense safely’	**Pharmaceutical expertise** Reliable and caring (*acting meticulously and carefully*) **Responsibility to society** (*guaranteeing continuity of pharmaceutical care by collaboration with other health professionals*) **Commitment to the patient's well‐being**
‘On the other hand, this patient, although living abroad, needed care’	**Responsibility to society** (*guaranteeing access to pharmaceutical care*) **Commitment to the patient's well‐being**

## Discussion

The pharmacy profession's core values were identified in moral dilemma narratives of pharmacists in community pharmacy. The descriptions of these values were customised for community pharmacy. All four professional core values, i.e. *commitment to the patient's well‐being*,* reliable and caring*,* pharmaceutical expertise* and *responsibility to society* were recognised in the dilemmas. As no other professional core values were identified, we believe the four core values are concordant with the practice of community pharmacy. The values *commitment to the patient's well‐being* and *reliable and caring* were prominent in almost all narratives. This suggests that pharmacists’ value patient‐centredness and concomitantly have retained their traditional attitude that emerges from Good Manufacturing and Good Clinical Practice. It confirms findings of studies among pharmacists in other countries.^2^, ^29^, ^31^ This study also reveals that pharmacists are aware of their *responsibility to society* as shown in their moral dilemma narratives. Although worldwide pharmacy associations emphasise the importance of responsibility to society,^21^ only in a few studies among pharmacists is this value reported.^2^, ^27^, ^31^

The strength of this study is that moral dilemmas were used that were actually experienced by pharmacists in community pharmacy practice. This study also has limitations. Firstly, the moral dilemmas were reported by PharmD students and ‘early career’ pharmacists whose recent training stimulates a patient‐centred attitude. These pharmacists may be more committed to patients’ well‐being because of more advanced training on their care role and the concerns and needs of patients, compared to earlier generations of pharmacists whose training focused more on caring for the product. Moreover, the training provided might have influenced their sensitivity for reflecting on the professional core values. However, it has also been reported that early career pharmacists might be more rule‐oriented even when this is not in the patient's best interest.^32^ Furthermore, research among senior community pharmacists is therefore recommended. Secondly, in the majority of the moral dilemma narratives, the outcomes were not clearly described. The actual weighing of professional core values could therefore not be analysed. As none of the professional core values is superior, it will be relevant to know to what extent the context of a moral dilemma influences the way pharmacists let one of the core values prevail in practice. Thirdly, the findings are confined to a Dutch context.

The core value *commitment to the patient's well‐being* is not limited to ‘what is best for the patient’ from the perspective of the pharmacist. Patients have their own health logic, preferences and values that pharmacists must consider. The ‘other value’ *protect life* highlights that pharmacists can also be motivated by personal or religious convictions. For example, pharmacists may decline to provide pharmaceuticals for euthanasia. Such convictions may conflict with professional core values and subsequently cause moral distress.^33^ To avoid moral distress, pharmacists could proactively make agreements to refer patients in need of euthanasia to other pharmacists who have no such convictions. Conflicting values and moral distress also occur among other health professions. Unfortunately, effective strategies to deal with moral distress have not yet been developed.^34^

The customised core value *reliable and caring* reflects that pharmacists not only felt a responsibility for the quality of pharmaceutical products, but also a responsibility to foster their professional relationships both with patients and other health professionals. It has previously been identified that the health professional‐patient fiduciary therapeutic relationship^9^, ^35^, ^36^, ^37^ as well as effective collaboration with other health professionals^38^, ^39^ are essential to (pharmaceutical) health care and can improve its services. However, aiming to care simultaneously for both these relationships often leads to moral dilemmas.^30^

The moral dilemmas in this study illustrate the complexity of the core value *responsibility to society*. The philosophy behind the current Dutch healthcare system is based on well‐known international principles: access to care for everyone, solidarity through an obligatory and accessible health insurance policy for all and good quality of care. The spending on pharmaceuticals is on the lower end of European and other Western countries. Pharmacists are expected to contribute to the sustainability of access to medicines by advising prescribers on cost‐effective prescribing and generic substitution of expensive specialties as much as possible. In some of the narratives, pharmacists are confronted with costs that will affect the sustainability of the pharmacy in such a way that *guaranteeing access to pharmaceutical care* for other patients will be endangered. For example, pharmacists reflect on the option to no longer freely dispense medication to patients who repeatedly cannot pay for expensive medication or on the decision to deliver additional care activities that are not reimbursed. The financial concerns/business pressure have been reported by several studies.^2^, ^31^ However, the narratives in our study did not obviously show commercial behaviour. For example, we did not come across narratives wherein pharmacists described situations in which they were more focused on selling (more expensive) products than trying to provide the best pharmaceutical care to patients. Pharmacists, like all other health professionals, need to distinguish between healthcare practice values and business values. For pharmacists in community pharmacy, this may even be more complex, as the public often perceives them as ‘shopkeepers’. For example, when a pharmacist proposes an elderly patient to start gastro‐protection because the patient receives an NSAID, the patient can perceive this proposal as a ‘selling practice’ by the pharmacist. However, this proposal is in accordance with clinical guidelines and pharmacists’ responsibility towards the patient (prevention of stomach bleeds) as well as to society (prevention of the costs of hospitalisation).

The dilemmas clearly show that the pursuit of the pharmacist to apply his or her pharmaceutical expertise to promote the appropriate use of medicines may conflict with other professional core values. In approximately half of the moral dilemmas, *reliable and caring* and *pharmaceutical expertise* played a role together with *commitment to the patient's well‐being*. Pharmacists experienced moral dilemmas because they could not apply their expertise when patients or other health professionals did not take them seriously.^30^ For example, pharmacists described that they could not provide appropriate pharmaceutical care because physicians did not listen to their pharmacotherapy suggestions. Similarly, this was the case when patients became aggressive or showed claiming behaviour in such a manner that this undermined the trust‐based relationship.

The findings of this study show that professional core values play a role in community pharmacy practice. The findings can be used to train pharmacists in recognition and reflection on moral dilemmas. Clear descriptions of professional core values can support community pharmacists in their daily practice. Recognition and reflection on professional values involved in moral dilemmas will help pharmacists to act in the best interests of patients.

The findings also may stimulate the dialogue on professional values of pharmacists in other sectors and worldwide.^[^ 6, 8, 9, 10, 14, 40, 41 This dialogue already exists in other healthcare practices, such as medicine^11^, ^12^, ^42^, ^43^, ^44^ and nursing.^45^ Common in these dialogues is the importance of shared professional values. This stimulates the development of a common professional identity.^12^ In contrast with physicians and nurses, pharmacists’ identity is still often perceived by consumers (as well as policymakers)^46^ as dual: that is, simultaneously being care professionals as well as entrepreneurs.^10^, ^13^, ^46^ Health professionals, acting on the basis of shared professional values that aim to serve patients and the public, do justice in fulfilling the social mandate of that practice^9^; it stimulates a shared accountability.^12^ Patients and the public are more inclined to trust such professional practices. Pharmacy associations should raise awareness among policymakers, regulators and educators on the importance of the societal embeddedness of pharmacy practice.^41^, ^46^ All these stakeholders should equally understand pharmacists’ societal role and contribution. Each country should, however, work out the professional core values themselves as (community) pharmacists’ societal role and responsibilities vary per country.^3^, ^10^, ^21^

## Conclusion

Professional core values were identified in moral dilemma narratives of pharmacists in community pharmacy and customised for their practice. All previously defined professional core values (i.e. c*ommitment to the patient's well‐being, reliable and caring, pharmaceutical expertise* and *responsibility to society*) played a role therein. The customised core values can enable pharmacists to better recognise moral dilemmas in practice. This can add to the advancement of the profession as a pharmaceutical care practice.

## Declarations

### Conflict of interest

The Author(s) declare(s) that they have no conflicts of interest to disclose.

### Funding

The authors received unrestricted grants from the Royal Dutch Pharmacists Association and from the foundation ‘Stichting Management voor Apothekers en voor de Gezondheidszorg’ (MAG).

### Authors’ contributions

M Kruijtbosch is the first author. She and W Göttgens‐Jansen were involved in data collection. All authors were involved in the study design, analysis and interpretation of the data, as well as article write‐up. All authors share full responsibility for the final content of the article and state that they had complete access to the research data that were used. All Authors state that they had complete access to the study data that support the publication.

### Ethical approval

We requested the Medical Ethics Review Committee of the University Medical Centre Leiden for ethical approval of the study. In a reaction to this request, the committee sent us an e‐mail (20 April 2016) wherein they stated that the Dutch Medical Research Involving Human Subjects Act (WMO) was not applicable for our study. Ethical approval, therefore, was not required.
